# Advancing behavioural genomics by considering timescale

**DOI:** 10.1038/s41467-018-02971-0

**Published:** 2018-02-12

**Authors:** Clare C. Rittschof, Kimberly A. Hughes

**Affiliations:** 1 Department of Entomology, University of Kentucky, Lexington, KY 40546 USA; 2 Department of Biological Sciences, Florida State University, Tallahassee, FL 32306 USA

## Abstract

Animal behavioural traits often covary with gene expression, pointing towards a genomic constraint on organismal responses to environmental cues. This pattern highlights a gap in our understanding of the time course of environmentally responsive gene expression, and moreover, how these dynamics are regulated. Advances in behavioural genomics explore how gene expression dynamics are correlated with behavioural traits that range from stable to highly labile. We consider the idea that certain genomic regulatory mechanisms may predict the timescale of an environmental effect on behaviour. This temporally minded approach could inform both organismal and evolutionary questions ranging from the remediation of early life social trauma to understanding the evolution of trait plasticity.

## Abstract (web summary)

Gene expression and behaviours are intimately related, and their interactions can play out over timescales from developmental to evolutionary. Here, the authors review how temporal aspects of gene expression mediate behavioural responses to the environment, a key question in behavioural genomics.

## Introduction

**Fig. 1:**
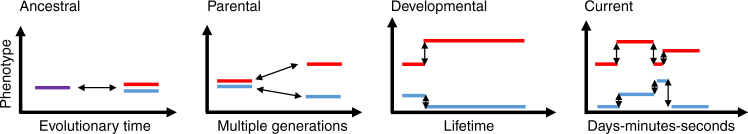
Environmental inputs at different time points (Ancestral, Parental, Developmental and Current) have variable timescales of effect (Evolutionary time, Lifetime and Days-minutes-seconds). The ancestral environment (e.g., the ecological context and selection pressures faced by individuals, including resource abundance, competition and predation threat) is transmitted across generations genetically as sequence level variation, and thus is considered as part of a spectrum of environmental circumstances (i.e., abiotic and biotic factors) that impact behavioural expression^65^. In the hypothetical example above, a behavioural phenotype is influenced by (left to right): (1) inherited gene sequence variation that evolved over time (purple vs. blue and red), (2) parental influences (which may include chromatin-based epigenetic effects, or other features under parental control, e.g., egg composition or oviposition site), (3) the environment experienced throughout development (including impacts on tissue structure or other mechanisms), and (4) the current environment experienced in real time. Solid black arrows indicate shifts among levels of a phenotype. We propose that timescales for behavioural effects are likely non-independent due to shared underlying regulatory mechanisms

Over a lifetime, behaviours are shaped by a vast number of external inputs and the internal state of the animal; timing is a critical component of this process. For example, experiences like predation threat or social stress elicit rapid behavioural responses, but also influence the physiological properties of the organism including metabolic rate, immune function and brain structure^1–4^, which can have behavioural effects over longer time horizons. Indeed, each external influence on behaviour has its own timescale, i.e., a timespan over which the input perturbs an organism’s internal processes with behavioural consequences (Fig. 1). Some experiences exert lasting effects on behaviour, while other effects are relatively transient^5,6^. In some cases, impacts on behaviour may manifest only later in life, or only under certain conditions. This behavioural plasticity within a lifetime, integrated with the evolutionary history encoded in the genome, has important fitness consequences for an individual^7^. However, the persistence of environmental effects over time can make it difficult to interpret the adaptive significance of behavioural plasticity in a given ecological context; the response to new inputs may be constrained by an organism’s earlier experiences. This outcome is particularly likely if the same underlying mechanisms that regulate a behaviour are influenced by disparate environmental inputs over time. In these cases, ancestral environments, parental effects and early-life environments experienced during development may have a combined influence on an organism’s behavioural response to the current environment (Fig. 1). The goal of this review is to consider behavioural dynamics in the context of the timescales of environmental influences, specifically whether certain categories of regulatory mechanisms predict temporal properties of environmentally responsive behavioural phenotypes. Defined broadly, regulatory mechanisms could include changes in genome function, hormone signalling, protein expression or tissue structure.

**Fig. 2:**
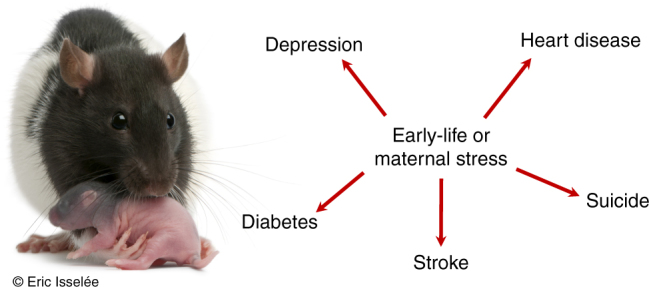
The early-life environment, including maternal care behaviours, have broad and sometimes long-lasting health outcomes in mammals including humans. Stress reactivity is one iconic example of the effects of maternal stress on offspring behavioural phenotypes later in life. Rat with pup, photo credit: Eric Isselée/Shutterstock. All rights reserved

While using mechanistic information to predict behavioural plasticity or stability is of clear interest to organismal biologists, such information is also important to evolutionary biologists. Natural selection presumably favours the ability to integrate and weigh environmental inputs from different sources, but how is such an ability optimized when inputs from different moments in time act through shared mechanisms, and thus have non-independent effects on behaviour?^8^ For example, in rats, stress reactivity is environmentally sensitive early in life, even to minutes-long environmental threats, with lasting impacts in adulthood due to the existence of a singular stress response mechanism (hypothalamic−pituitary axis reactivity, Fig. 2, Box 1). These impacts in adulthood, however, are only adaptive under certain environmental conditions (Box 1), which means that early-life experiences entrained via relatively stable mechanisms (e.g., changes in genome regulation) may give rise to environmental mismatch, a cost of plasticity, particularly in a rapidly fluctuating environment. Few studies have considered the consequences of mechanistic non-independence in terms of the evolution of gene−environment interactions and phenotypic plasticity^1,9–11^. We propose that the type of mechanism that regulates a behaviour may affect the likelihood that plasticity will evolve; conversely, selection for plasticity could determine the nature of underlying regulatory mechanisms.

Predicting timescales of environmental influence using information about underlying regulatory mechanisms is not entirely new. For example, in the context of memory stability, transient increases in neurotransmitter release underlie short-term memories, while long-term memories require new protein synthesis^12^; these distinct molecular mechanisms define the timescale of a memory and thus theoretically could be used to predict a memory’s longevity. Similar types of predictions arise in the field of behavioural genomics, which explores how gene expression dynamics correspond to and predict behavioural variation within and among individuals. We focus this review on common hypotheses about genomic regulatory mechanisms and their links to behavioural change. For instance, it is hypothesized that events that lead to changes in brain gene expression result in relatively stable changes in behaviour compared to those that do not^13–16^, and that experience-induced chromatin modifications are associated with even more stable shifts in behaviour^17,18^. Few studies, however, have explicitly evaluated these hypotheses by characterizing the temporal properties of experience-induced behavioural expression. Moreover, there are few general principles to predict how behavioural phenotypes will be expressed when multiple environmental inputs converge on a similar genomic mechanism over time. We evaluate the state of knowledge in these areas, and suggest future studies that could more robustly assess the relationship between gene expression plasticity mechanisms and behavioural temporal dynamics. Our primary focus is to apply the principle of timescale in the context of behavioural phenotypes because they are unique both in the complexity of their genetic regulation and their degree of environmental responsiveness. Notably, the principles described herein may also be relevant to other rapidly changing, non-behavioural phenotypes, which we revisit in the Summary and Future Directions section.

## Gene expression as a focal timescale mechanism

Though there are many types of mechanisms that underpin behavioural variation (e.g., neurotransmitter receptor numbers, neuronal connectivity, endocrine function, and morphology and tissue structure^19^), here we narrow our focus to gene expression dynamics and their regulation. Variation at other mechanistic levels has important temporal correlates, but gene expression dynamics are easy to measure, and they show plasticity across a very broad temporal range (see ‘Timescales and genomic correlates of behaviour’ section below). Furthermore, gene expression measures allow for a range of analytical approaches, from a focus on the dynamics of a specific pathway to plasticity at the whole-genome scale, agnostic to gene identity. This range enables high-powered statistical approaches.

A change in genomic state (i.e., cellular or tissue level patterns of gene expression and gene regulation) is a particularly popular method to infer that an environmental input causes a lasting behavioural change^16^. This inference is based on the assumption that, unlike transient electrical signals in the brain, changes in gene expression will translate to lasting shifts in protein expression and higher level physiological changes^13,20^. Although some studies show correlations between trait stability and the degree of differential gene expression^15^, one surprising insight from behavioural genomics studies is that gene expression changes co-occur even with relatively ephemeral environmental inputs and behavioural shifts; moreover, these expression changes are widespread throughout the genome, including changes in regulatory genes as well as their downstream targets^21^. Because gene expression variation is associated with behavioural change at a range of different timescales, it offers an experimentally tractable level of organization to assess behavioural temporal dynamics and to determine mechanistically how environmental inputs over time interact at the genomic scale to influence behavioural phenotypes. Moreover, because genomic state is a biochemical property, there may be general rules to its modulation and underlying regulatory mechanisms that apply broadly across divergent species, despite other difference in brain morphology and physiology^22–24^.

**Fig. 3:**
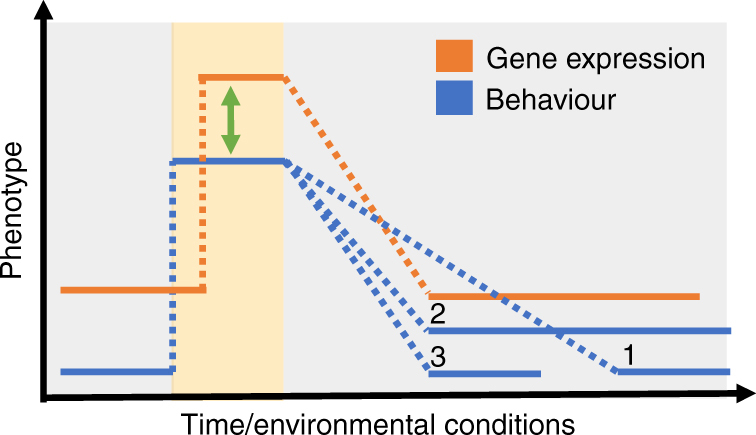
Gene expression and behavioural dynamics are understudied, but may provide insights into other levels of organization that influence behaviour. Changes in phenotype in response to a transient shift in environmental conditions (grey vs. beige) is indicated as an increase in gene expression and behaviour. In this figure, we arbitrarily chose to show the direction of change for both phenotypes as an increase. The direction of any given behavioural change is context-dependent, and for many behaviours, genes show changes in expression in both directions (notably some genes, e.g., immediate early genes, commonly increase in expression^22^). The green arrow indicates the potential interaction between behaviour and gene expression. Hypothetical outcomes of this interaction (blue lines 1–3) are discussed in Box 2

It is important to note that although the co-occurrence of gene expression variation and behavioural variation suggests an interaction between these levels of organization^10^, the relationship is complex and likely indirect^21,25^, and both phenotypes are to some degree independent of one another. Furthermore, the direction of causation between behaviour and gene expression change is unclear and under-investigated. Though these are general issues for linking many levels of physiological organization to behaviour, the problem is particularly acute for genomics research, where studies often show surprisingly large changes in gene expression with limited causal validation. These facts present important challenges to using genomic state to predict timescale of behavioural change, or vice versa. In the current review, we explore the use of gene expression analyses to predict timescale of behavioural changes, assuming at least some direct relationship between expression values and behavioural trait values. However, in Box 2 (Fig. 3), we explicitly propose some hypothetical patterns for gene expression and behavioural variation that may point towards changes or stasis at other mechanistic levels of organization. Such hypotheses will help direct future investigations into the nature of the relationship between gene expression and behavioural variation.

In the following section, we highlight diverse cases in which behavioural change over different timescales, both within the lifespan of an organism and across generations, is correlated with variation in expression of similar sets of genes. These patterns, which are observed with increased regularity, provide a basis for the argument that mechanisms of behavioural variation with respect to time are non-independent, and that gene expression reflects the integration of information from ancestral environments (i.e., gene sequence level variation) with parental, developmental, and current environment effects (i.e., acute stimuli that activate or modulate transcription) using a single currency.

## Timescales and genomic correlates of behaviour

One generalized finding from behavioural genomics is that even relatively ephemeral shifts in behavioural phenotype are accompanied by widespread changes in the expression of hundreds and even thousands of genes in tissues including, but not limited to, the brain^26^. Studies have examined gene expression signatures associated with behavioural variation across a range of time points, including over evolutionary time where sequence level variation underlies behavioural variation across distinct species and populations^27^, cases in which cues from the environment are transmitted from parents to offspring^28–30^, developmental time, during which cues shape both behaviour and morphology^31^ and current environmental time, in which acute interactions with conspecifics, predators, or pathogens influence behaviour^32^. The widespread co-occurrence of variation in behavioural and genomic states across different points in time and with different timescales of effect suggests gene expression is a relevant mechanistic level to examine how environmental inputs are integrated to give rise to behavioural phenotypes.

A number of studies have found that patterns of differential gene expression associated with phenotypic or behavioural traits indeed involve similar sets of genes regardless of the timescale of effect, providing evidence that molecular mechanisms of behavioural change are non-independent. Many studies have identified mechanistic links across ancestral and more proximate timescales. For example, investigations of genetic accommodation, in which plasticity in a given trait or expressed gene product precedes evolved differences that reflect sequence level variation^33^, have found overlap in gene expression associated with heritable variation and within-population plasticity in the same trait^34^. One of the earliest cases of gene overlap across ancestral and current environmental time for a behaviour involved the *foraging* gene, for which sequence level variation in the fruit fly (*Drosophila melanogaster*) influenced both gene expression and behavioural differences across individuals with different genotypes (ancestral timescale), while regulatory changes over the course of adulthood (over a timescale of weeks) gave rise to similar effects in the honey bee (*Apis mellifera*)^35^. In killifish, genes showing population level expression differences correlated with variation in salinity tolerance were also responsive to acute osmotic shock^36^. In sailfin mollies, there is substantial overlap between genes differentially expressed as a result of plastic vs. genetically fixed differences in male mating strategies^37^.

**Fig. 4:**
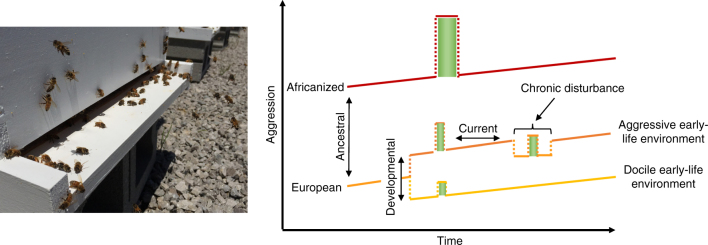
Honey bee aggression shows a high degree of environmental sensitivity throughout life, as well as heritable variation as a function of genotype. Right: Solid lines indicate the continuous increase in aggressive tendency that occurs as bees age. Dotted lines indicate environmentally induced changes in aggression, which have variable timescales of effect. Green bars indicate short-term changes in aggression as a function of acute exposure to a predator threat. Left: Active honey bees at the colony entrance (Photo by C. Rittschof)

Fewer studies have evaluated mechanistic overlap for environmental inputs received at different time points within a single individual’s lifespan (i.e., outside of ancestral effects)^11,38^. This could include cases in which developmental regulatory mechanisms are co-opted later in life to regulate plastic phenotypes, a phenomenon that has been demonstrated in the context of neural plasticity^39^. In the zebra finch, genes that are constitutively expressed during song learning periods are largely suppressed during adulthood, which is outside of the critical period for song learning^40^. However, these genes can become activated in response to a strong social stimulus, even during adulthood^40^. Box 3 discusses a similar phenomenon investigated in detail and across multiple contexts for honey bee aggression (Fig. 4).

Despite considerable evidence for shared gene expression patterns across timescales, some studies have revealed interesting exceptions and complexity in the nature of transcriptional change. For example, in guppies, an evaluation of the brain transcriptional response to predator exposure during development reported that genes that evolved rapidly over evolutionary time were the same as those showing plasticity in response to predator exposure during development; however, the direction of plasticity induced by predator exposure was largely opposite in direction to their evolutionary trajectory. Thus, the transcripts that were revealed to show ‘non-adaptive’ patterns of plasticity during development were also the first to evolve changes in expression, presumably because non-adaptive plasticity would be subject to very strong negative selection^41^. A recent study of *D. melanogaster* genomic response to dietary stressors yielded similar results, with overlap in 108 genes differentially expressed as a result of experimental evolution vs. acute exposure to a specialized diet. Similar to the guppy example, over 90% of these overlapping genes were expressed in opposite directions across timescales (‘membrane’ and ‘transmembrane’ were identified as significant functional categories enriched for genes in this analysis)^42^. These studies, notable because they are experimental and not correlational, raise the possibility that patterns of gene expression evolution may be a function of evolutionary divergence time, where early divergence is characterized by non-adaptive plasticity (which is strongly selected against until it is purged), while divergence in later generations reflects the patterns expected if adaptive plasticity facilitates evolutionary change^38,42^. Also known as ‘counter-gradient selection’^42^, another explanation for this pattern is that evolutionary and induced gene expression effects combine to yield little net change in phenotype, suggesting a set-point (possibly constrained by physiological demands of the organism) that is maintained by integrating evolutionary history with current environmental conditions. Further experimental tests of ‘early-non-adaptive, late-adaptive facilitation’ may be a useful empirical starting point for gaining a better understanding of molecular plasticity mechanisms and their evolution^41^. These patterns for molecular plasticity are particularly intriguing given positive relationships between trait plasticity and trait evolution for other phenotypes evaluated in experimental studies^43^.

Gene expression dynamics are a function of underlying regulatory mechanisms that could constrain or predict capacity for plasticity at both the gene expression and behavioural levels. In the next section, we review some major mechanisms of transcriptional regulation, including ones often invoked to predict stability in gene expression and behavioural state. We discuss the known temporal rules associated with these mechanisms in order to assess the state of knowledge in the field, but also to evaluate whether this type of information could provide a means to predict the time course of experience-dependent changes in behaviour.

## Timescales of gene expression regulatory mechanisms

Many studies use the existence of particular gene regulatory mechanisms to infer the relative stability of an experience-induced behavioural change. One common example is the evaluation of epigenetic modifications (e.g., changes in DNA methylation), which are used across species from insects to mammals to infer stable shifts in gene expression and behaviour^17,18^, despite substantial differences among taxa in the prevalence and function of these modifications^44^. An extremely broad array of gene regulatory mechanisms entrains environmental inputs to influence mRNA levels and gene transcription. Determining whether these mechanisms have predictive value in terms of the timescale of effect, i.e., the relative stability of the effects on the transcriptome has been emphasized recently as a controversial yet critical area of research in a number of different plasticity contexts, from learning and memory to transgenerational inheritance of environmental effects^45–48^. Here we systematically review some of the major types of gene regulatory mechanisms and what is known about their temporal stability and reversibility. Although these mechanisms could differ across taxa in terms of their prevalence or function^49^ (see also below), here we cover broad taxa in general terms because there is a lack of evidence for taxon-specific differences in the temporal properties associated with these regulatory mechanisms.

A number of relatively understudied regulatory mechanisms may play critical roles in modulating gene expression and behaviour, especially over relatively short-term timescales. Temporary inductions in gene expression may be maintained passively for a period of time depending on the rate of mRNA decay^50^. Experience-induced variation in gene expression can also be maintained by self-sustaining feedback loops in which protein concentrations modulate mRNA transcription^46^. Such mechanisms have been known to play a role in circadian rhythms^51^, hormone signalling more generally^52^, and energy metabolism and metabolic capacity^53^. Functional genomics studies have linked shifts in all three of these biological processes to variation in behavioural phenotypes^38,54–57^; thus it seems likely that mechanisms of autoregulatory transcriptional control could play an important role in modulating experience-induced behavioural change. However, because elucidating such mechanisms is difficult to do in a high throughput manner in naturally behaving organisms, there is limited knowledge of the temporal resilience of these regulatory mechanism, and their prevalence.

Chromatin-based transcriptional regulatory mechanisms, e.g., DNA methylation and histone modifications, have received a large amount of attention in a variety of disciplines^20,46,47^. These mechanisms have been associated with environmentally induced behavioural variation across generations^31,58^, during development^49^, and throughout adulthood^17,45^. When considering the timescale of changes in behaviour and gene expression, one simple heuristic is that stimuli that result in lasting behavioural shifts will be those that cause chromatin-based epigenetic changes. However, though it has been demonstrated that these types of changes can lead to stable shifts in gene expression that last for substantial portions of an organism’s life^59^, there is limited understanding of the reversibility of these mechanisms, both in terms of the time course and the relevant stimulus inputs^17,60^. For example, in mammals, contextual fear learning requires a combination of active DNA methylation and demethylation^45^ to tune gene expression patterns in neurons. In the honey bee, some of the most highly methylated genes show the greatest degree of context-dependent plasticity^61^. The assumption of DNA methylation persistence over time in particular is at least in part historical, since this mechanism was first appreciated in the context of stable maintenance of cell identity across cell divisions^46,58^. Interestingly, over some of the longest timescales for behavioural change, e.g., transgenerational inheritance of parental effects, organisms undergo known periods of epigenetic reprogramming that may essentially erase experience-based effects on gene expression^46^; thus, a simple relationship between the duration of an environmental effect and chromatin-based regulatory mechanisms may lead to spurious conclusions.

New research continues to alter the perspective on the time course and reversibility of epigenetic modifications, particularly in natural contexts that involve repeated and varied environmental inputs throughout life^58^. One feature that makes the study of these mechanisms and their temporal components so challenging is that the occurrence and the outcomes of chromatin modifications (whether they upregulate or downregulate gene expression, or cause other types of changes, e.g., changes in mRNA splicing) are extremely varied across taxa^62–64^. In addition, several types of RNAs have been implicated in transcriptional regulation, independently and in conjunction with other epigenetic modifications. For example, small RNAs and long non-coding RNAs can modulate other regulatory mechanisms including chromatin modification^46,65–68^. Rapid and reversible shifts in behaviour can be difficult to study in any context, which may explain the limited application of chromatin-based analyses to short-term timescales. However, there are many experimental tools that can be used to manipulate and measure chromatin-based modifications towards a better understanding of their temporal effects on gene expression^69,70^.

Regardless of the mechanisms responsible for maintaining stability in environmentally induced shifts in gene expression, a large knowledge gap with respect to behaviour is simply an understanding of the temporal dynamics of gene expression changes following a stimulus, and specifically, the relationships among these dynamics, behaviour and modulation at intermediate levels of organization (e.g., protein levels, brain structure^14,71,72^) (Box 2). It is well-known that different genes show different activation timing in response to an environmental stimulus (e.g., immediate early genes) in part because of their positions in larger networks. Less is known about the arc of the gene expression response over time, especially in the context of repeated stimuli that may activate the same set of genes, and in light of the fact that mRNA levels are subject to noise and stochasticity^73–75^.

Notably, there is evidence that activational transcriptional responses to acute stimuli follow similar rules across species^22,23^. By extension, it may be possible to determine general rules for transcriptional dynamics, and moreover, how these dynamics are linked to behavioural expression, higher levels of biological organization that modulate behavioural phenotypes, and the capacity for and evolution of behavioural plasticity.

## Timescales and the evolution of behavioural plasticity

A plasticity limit is a type of constraint on the evolution of phenotypic plasticity^76^. A plasticity limit refers to an inability to achieve an optimal trait value, often due to the temporal properties associated with switching among trait states^76^. Examining plasticity limits at the biochemical level (including genomic state) may provide insights about plastic phenotypes and their evolution^9^. For example, animal personalities (consistent differences among individuals in behaviour) can arise if environmentally responsive behavioural phenotypes are stabilized over time through their dependence on slower changing intrinsic state variables^77^. Genomic state, as an underlying intrinsic state variable, could thus impose a limit to plasticity in some cases. For instance, in the honey bee, chronic exposure to predator disturbances leads to baseline differences in both aggression and brain gene expression^78^, and this shift persists for several days after the threat is removed. One hypothesis to explain this phenomenon is that the behavioural response to the repetitive predator stimulus persists until the gene expression response decays, suggesting the behaviour is stabilized because the genomic state shifts slower than the external environment. The result is that behaviour−environment match at a given time point is limited by previous experiences that influence both behaviour and genomic state (a form of ‘plasticity-history limit’ to plasticity evolution)^9^.

In some cases where individuals can shift phenotypes bidirectionally, or multiple times within their lifespan, transitions are asymmetric, occurring more rapidly or more easily in one direction than another (a form of ‘lag time limit’ to plasticity evolution). Asymmetry can result in a temporary mismatch between environment and phenotype, a limit to plasticity evolution^9^. Studies suggest this type of plasticity limit may have generalizable gene expression characteristics. For example, in *Locusta migratoria*, which can transition between a solitary and gregarious morph, increased density and the resulting heightened interaction between conspecifics initiates the transition to the gregarious morph^28,79^. Repeated stimuli are required to complete this physiological and morphological transition. The switch to the gregarious morph is relatively slow compared to the reverse despite the fact that this transition is accompanied by more robust changes in gene expression^33,34^. This robustness could be the result of repeated stimulus exposure over an extended timeframe, compared to the shift towards the solitary state, which is initiated by the absence of stimuli^80^. Thus, the degree of gene expression change is a function of the nature of the stimuli that cause the phenotypic shift, not the rate or degree of the phenotypic shift itself. In this example, there are limits to plasticity associated with a time lag in the phenotypic shift, which may result in fitness costs associated with environmental mismatch. The tolerance of these asymmetric time lag limits over evolutionary time is thought to reflect the higher fitness costs associated with transitioning to the gregarious morph in error. However, it is important to consider the relationship between asymmetric limits and the mechanisms that regulate gene expression changes. If stimuli frequency, gene expression patterns and the speed of phenotypic transition are typically linked, type and frequency of salient stimuli, as well as gene regulatory dynamics, could be used to predict plasticity limits and potential costs to plasticity evolution. Conversely, selection could shape the nature of underlying gene regulatory plasticity mechanisms.

Even when phenotypic transitions are symmetric, important differences in underlying mechanisms could reveal hidden direction-specific constraints. *Astrotilapia burtoni* is a cichlid fish in which males can transition between dominant and subordinate states that differ in behaviour, body colouration and testis size. Behaviour changes very rapidly regardless of the direction of transition^19^. Studies examining a small set of transcription factors showed that the same transient increase in immediate early gene expression is involved in both ascent to and descent from dominance. However, brain regional patterns of gene expression differentiate the direction of social transition^20,81,82^. Thus, similar genes are used in transition and expressed with the same temporal rules, but the location of expression is direction specific. Altering the regional location of variation in brain genomic state could differentially impact other phenotypes regulated by those same brain regions, perhaps imposing a cost to plasticity that extends beyond the focal behavioural phenotype.

The ‘epiphenotype problem’^9^ is a plasticity limit that occurs when environmental inputs occur following the window of a developmental critical period. In the case of morphology, if these inputs occur late in the development of a trait, they may not have the capacity to fully alter the developing structure. Similarly, for behaviour, late inputs may not be robustly assimilated because major periods of brain development have lapsed. The idea of critical periods may also apply in important ways in the context of gene expression. Developmental time periods are critical periods for chromatin modifications in some cases^11^, and inputs during these periods may set the capacity for additional plasticity^8^. Such epigenetic processes, however, are also hormonally responsive throughout life, and particularly plastic in the nervous system^11^. More work is needed to determine, for example, whether periods of hormonal shifts (e.g., during the sexual maturation process) may serve as additional critical periods for epigenetic reprogramming, with temporal consequences. These possibilities suggest the epiphenotype problem may apply beyond morphological contexts, and could be considered a limit in the context of behavioural plasticity.

## Future directions

Behaviour presents unique challenges to phenotypic plasticity theory owing to its exceptional capacity for change. However, behavioural plasticity is far from unlimited, due at least in part to substantial variation in the timescales of environmental effects on behavioural expression. Here we explored evidence for the idea that genomic mechanisms that entrain environmental experience at different timescales are non-independent. If so, the temporal properties of the mechanisms that regulate gene expression could be used to predict the relative stability of environmental effects on behaviour. This is especially true if gene expression dynamics track behavioural dynamics and reflect the integration of one or several environmental inputs. Finally, from an evolutionary perspective, mechanistic knowledge may uncover the characteristics of and ultimately predict circumstances under which individuals integrate or prioritize of environmental inputs over time. However, there remain a number of outstanding questions and experimental challenges that are limiting our understanding of temporal rules of genomic regulatory mechanisms, how information from environmental cues is integrated over time, and how a focus on individual behavioural traits can be expanded to consider multiple correlated phenotypes.

### Limited evidence for temporal rules for gene expression regulatory mechanisms

While there is reasonably strong evidence for overlapping, non-independent patterns of gene expression across timescales of behavioural variation, evidence that specific regulatory mechanisms encode time and thus can be used to predict gene expression temporal dynamics is lacking. More experimental evidence is needed to link certain types of regulatory mechanisms (e.g., chromatin modifications) to particular time frames for stability in gene expression and behaviour^11,46^. The intuitive hypothesis that relatively high-stability behavioural phenotypes are underpinned by chromatin modifications, while more transient shifts in behaviour are associated with more labile expression mechanisms may not be supported, at least in this most simplistic form.

Fortunately, with the increased capacity to generate transcriptomic data, it is possible to evaluate the temporal component of gene expression and behavioural dynamics simultaneously in order to gain a better understanding of how plasticity at these two levels is linked. This information could then be used for more targeted assessment of regulatory mechanisms associated with highly labile vs. more stable patterns of behaviour and gene expression. For example, in the honey bee, genes involved in energy metabolism are highly methylated, but also highly responsive to environmental inputs throughout life (e.g., in the context of aggression, Box 3)^61^, which raises the possibility that methylation may not necessarily confer stability. Assessing first the temporal dynamics of gene expression and behaviour, and then assessing regulatory mechanisms for target genes may provide more meaningful insights about regulatory mechanisms that confer gene expression stability. A second approach could be to explicitly test the reversibility of presumed long-lasting or stable behavioural phenotypes and gene regulatory mechanisms^30,59,83^. For instance, only in certain cases have the reversibility of social experiences during developmental critical periods been investigated, particularly for social effects on the genome.

We argue that in addition to generating –omics data to assess epigenetic marks and their behavioural correlates, there is value in focusing explicitly on gene expression temporal dynamics^74,75^. Notably, although RNAseq data is easy to generate, it is still expensive to collect at the sample sizes necessary for behavioural experiments, since gene expression associated with behavioural change is characterized by a relatively high degree of individual variation and small effect sizes^84^. Adding a temporal dimension to data collection may be cost prohibitive in some cases (but see Lohman et al.^85^). However, faced with a monetary trade-off between generating transcriptomic data at multiple time points and other –omics data that address underlying regulatory mechanisms, e.g., bisulfite sequencing data to examine patterns of DNA methylation, temporal expression data, even for a subset of genes, may be more valuable to the questions posed herein.

### Experimental and informatics approaches to evaluate gene expression temporal dynamics

In addition to monetary considerations, there are important analytical challenges to assessing temporal dynamics of gene expression and behaviour. For one, gene expression patterns are highly dependent on sampling time point, and it can be difficult across organisms and behavioural contexts to select a relevant sampling strategy. Moreover, because behavioural phenotypes are often polygenic and reflect epistatic interactions among loci, evaluating expression dynamics may require gene network-level data and analyses. For instance, network or epistatic relationships among genes can be malleable (e.g., with genetic background^86^), while in some cases, networks are relatively robust to perturbation. Exploring the relative robustness of gene expression networks may be a powerful tool however, if it reveals that certain subnetworks are more resilient to change than others. Such a finding could help narrow the focus to the most labile genes^87^. More simplified approaches that target candidate genes and pathways associated with certain behavioural traits may also prove insightful^10^.

There are some notable analytical considerations with respect to identifying mechanistic commonalities across timescales (reviewed in ref. ^26^). It is inherently easier to find evidence of overlapping gene expression profiles across timescales than to say definitively that expression profiles are unique. This means that the ability to identify cases where completely unique sets of genes give rise to the same behavioural phenotype, the alternative hypothesis to non-independent mechanisms, is limited. Similarly, even in cases of gene expression overlap, there are always some genes that are context-specific, and it is not always possible to identify which genes are most critical to patterns of behavioural variation. Finally, certain genes may play discrete roles in modulating trait means and trait variability^88^, and the relationships between gene expression values and phenotypic change may also shift over the lifetime due to critical changes in regulatory interactions among genes^87^. All of these are important considerations when assessing patterns of genomic similarity across timescale.

Parsing the vast quantity of information from whole-genome transcriptional studies to establish causal relationships between genes and behavioural phenotypes^26^ and to determine the most significant predictive patterns with respect to timescale present major informatics and empirical challenges for the coming years. Causal relationships between gene expression and behaviour, and their associations with time, could be addressed with guidance from existing behavioural genomics data sets, in which there are numerous cases where genes from measurable physiological pathways are tied to behaviours that are plastic across variable timescales^10,14^. Relatively simple causal experiments that perturb these pathways pharmacologically or genetically could be used to assess how gene expression dynamics for subnetworks of interconnected genes correspond to behavioural dynamics over time.

### Consideration of non-behavioural phenotypes

Temporally linking RNA abundance and behaviour may point to cases in which higher levels of biological organization (e.g., brain structure and connectivity) play an important role in modulating behavioural phenotypes with respect to time, especially if changes in brain structure or function manifest as a predictable mismatch between gene expression and behavioural dynamics (Box 2). Importantly, other levels of biological organization may show plasticity at a temporal frequency similar to behavioural phenotypes, suggesting that the framework we present here could be generalized to other types of traits. For example, physiological phenotypes, e.g., stress response, salinity tolerance (discussed above^89^), immune function^2^, and energy metabolism^90^ can show relatively rapid shifts over time, but are also subject to some of the same timescale properties as behaviour. Some of these traits, like immune function, are even activational in nature, similar to behaviour. Investigating the gene expression principles discussed herein in the context of non-behaviour phenotypes has the added value of addressing whether or not the nature of expression dynamics and regulatory mechanisms are specific to the brain, or if these are patterns that can be extrapolated to other tissues.

### Gene expression dynamics in models of information integration over time

A growing body of literature applies modelling frameworks to understand how organisms integrate environmental information from multiple time points^1^. However, few models in this area account for situations in which environmental experience not only affects the organism’s informational status, but also directly impacts lasting somatic traits of the organism, e.g., body size or strength^1^. Incorporating measures of genomic state, particularly if gene expression dynamics show predictable temporal patterns, could be a relatively straightforward way to track changes in somatic or physiological state, and thus integrate mechanistic data into these models.

Models could also consider mechanisms for information integration, and how the nature of these mechanisms could feedback to influence behavioural trajectories over time. For instance, development can serve as a critical period of environmental influence that irrevocably shapes an organism’s physiology and behaviour throughout life, even with additional real-time environmental information. Though this type of critical period is presumably a target of selection, it may also be a generalized form of plasticity constraint. Adding mechanistic data about temporal plasticity constraints may improve information integration theory, and more generally, theory about plasticity evolution.

### Implications of timescale for correlated traits

Here we have largely focused on independent behavioural traits, but gene expression mechanisms associated with timescale also have implications for correlated traits. Evolution in response to selection on a suite of correlated traits depends on genetic variation and covariation among the traits^91^. Genetic correlations can be sensitive to normal environmental variation^92,93^, as well as more exceptional contexts like inbreeding or stress^94–96^. This environmental sensitivity means that spatial or temporal fluctuation in genetic correlations could substantially influence the evolution of behavioural traits. If traits depend on shared molecular mechanisms, independent optimization of the two traits might not be possible, at least over short to intermediate evolutionary time periods.

## Conclusions

Work in behavioural genomics has laid the foundation to analyse sophisticated relationships between molecular dynamics and behavioural state at different timescales. Future work in this field could greatly benefit from even relatively simple experiments that more clearly define how the environment influences the expression of non-independent and behaviourally relevant sets of genes. Gene expression, like behaviour, shows a high degree of environmental sensitivity. Tracking changes at both levels across time may provide new insights into behavioural regulation at intermediate levels of biological organization, and provide predictive value about the relative stability of environmental influences on behaviour.

### Box 1 Timing of social stress and human health implications

In the social and health sciences, there is a growing focus on the physiological and behavioural impacts of the social environment, which can influence susceptibility to illnesses including stroke, heart disease and depression^96,97^. One vexing reality is that some of these social effects are easily remediated while others persist throughout life^30^. Understanding the timescales of genomic effects may point to interventions that interrupt detrimental social effects on mental and physical health^97^.

*Stress reactivity* is a phenotype that is associated with physical and behavioural health problems throughout life—depression, heart disease, diabetes, stroke and suicide. It is also informed by multiple sources of inherited and environmentally derived information^98^. Rat pups that are experimentally handled for a short time period (3–15 min) early in life receive high levels of maternal licking and grooming upon return to the nest, and as a result show a diminished level of stress reactivity later in life. Licking and grooming results in increased expression of stress hormone receptors, mediated by epigenetic mechanisms^47,96^, and resilience to stressful situations, e.g., exposure to novel environments. Longer-term (180 min), but still relatively temporary experimental handling and separation from the mother, in contrast, has opposite effects on stress reactivity, resulting in a chronic upregulation of the stress response systems and a fear of novelty^99^. Thus, similar types of stressors, with minor differences in severity relative to the lifespan, have context-dependent effects on stress reactivity. Moreover, maternal care behaviours, as well as pre-natal maternal stress exposure, can override heritable genetic variation in stress reactivity^96^. Stress experienced at all of these time points is mediated by the same hormonal mechanism, which allows for the non-independent integration of information from multiple sources.

### Box 2 Hypothesized patterns of gene expression and behavioural variation

A shift in behaviour in response to acute changes in environmental conditions is mediated by an electrical signal in the brain, and thus occurs nearly instantaneously. This signalling event also results in changes in gene expression that occur on the order of minutes to hours^22^. The temporal dynamics that describe behaviour and genomic state following a signalling event are complicated by the fact that these two levels of organization interact and are often bi-directional (indicated by the green arrow in Fig. 3). Changes in mRNA abundance could ultimately lead to changes in expression of key proteins and modifications at other higher levels of organization in the brain, including changes in neurotransmitter receptor number, shifts in metabolic pathway flux, or changes in dendritic arborization or synapatic connections^38,56,100^. Such changes could anticipate a new behavioural state, or reflect stimulus recovery and restoration of a baseline, pre-stimulus brain state^14^.

The timescale of response at both the behavioural and molecular levels could be used to study dynamics at other levels of organization. Simple transcriptomic experiments coupled with temporal analyses may be used to establish some general rules about transcriptional and behavioural dynamics. We hypothesize several scenarios for the temporal patterns of brain gene expression and behaviour following an acute stimulus, or as environmental conditions temporarily shift (Fig. 3, beige) and then shift back (grey): (1) Compared to brain gene expression patterns, behaviour may show a protracted return to the baseline state, or (2) maintain a consistent level that deviates from the original baseline. Either of these outcomes might suggest at least some degree of interaction between behaviour and gene expression, and alterations to intermediate levels of biological organization in the brain. A third possibility (3) is that behaviour shows a rapid return to baseline that parallels gene expression change. This could imply minimal interaction effects across the two levels, or that expression changes are related to recovering from a stimulus rather than causing a persistent change in future behavioural state. Experiments that compare the results of gene expression and behavioural phenotypes with repeated stimuli would also be informative, as they examine how experiences with different timescales interact at both the behavioural and genomic levels^25^.

The activity of the genomic state following a stimulus may not be uniform across the genome. For example, in stickleback males, a time course analysis of brain gene expression changes following acute exposure to a territorial intruder revealed that genes grouped into 12−13 clusters with specific temporal expression patterns. For example, there was a rapid but transient increase in the expression of genes associated with hormone function, but slower changes in genes involved in immune function, which did not peak until hours following the stimulus. Genes that shared network properties, e.g., transcription factors, also tended to cluster together, suggesting a relationship between network position and temporal dynamics^75^. Future studies could evaluate how these gene expression dynamics correlate with transient vs. stable shifts in behaviour.

### Box 3 Plasticity in honey bee aggression as an example of a behavioural phenotype with strong temporal properties

In the honey bee, aggression is a socially regulated individual behaviour that facilitates collective nest defence^101^. Bees differ in both aggressive tendency (indicated by solid lines, Fig. 4), as well as the level and stability of their rapid aggressive response to an acute predator cue (indicated by green boxes) across several timescales. Differences in ancestral environments have resulted in heritable genetic differences in aggressive tendency comparing Africanized and European sub-species of honey bees. There is experimental evidence that Africanized bees respond more severely to acute predator cues compared to European honey bees^102^, and anecdotal evidence to suggest that this response is more persistent in Africanized bees. For both sub-species, aggressive tendency also steadily increases as bees age (‘Time’ axis in Fig. 4 right). In European bees, experimental evidence shows that larval and pupal developmental environment influences response to aggressive cues during adulthood, and presumably baseline aggressive tendency^103^. Moreover, ‘current’ ecological context during adulthood influences baseline aggression and rapid response to predator disturbance in European bees: exposure to alarm pheromone or invasion threat leads to a temporary increase in aggression^104,105^, and chronic predator disturbance leads to a sustained decrease in aggressive tendency as well as decreased responsiveness to acute threat^78^.

In honey bees, there is ample evidence that shared sets of genes are modulated in association with aggression across a range of different temporal contexts for information acquisition^38,54–56,78^. Whole-brain transcriptomic data have been used to evaluate this possibility for different bee sub-species, older and younger adult worker bees, and bees exposed to alarm pheromone relative to control^38,54^. A small subset of biomarker genes derived from this larger data set show that chronic predator threat exerts similar influences on brain expression patterns and behaviour^78^. These biomarker genes span a number of functional categories including stimulus perception and central energy metabolism. Comprehensive time course data evaluating plasticity in gene expression relative to behavioural variation in response to acute cues are scarce, but a recent study found that gene expression is quite dynamic over the course of 2 h following an aggressive encounter, with a small subset of genes showing a consistent signature of the aggressive experience over time^74^. Interactions of genotype, developmental conditions, disturbance threat and rapid response to acute predator cues on aggression have not been thoroughly explored, though a recent study suggests some acute social experiences impact behaviour without impacting gene expression^25^.

Work in the honey bee indicates that variation in aggression in multiple contexts is associated with shifts in brain energy metabolism (reviewed in ref. ^57^), assessed at the transcriptomic^38^, metabolomic^54^ and enzymatic^38^ levels. Thus, this system also provides a unique opportunity to study how genomic state corresponds to functional changes at higher levels of biological organization that also influence behavioural plasticity.
